# A New Analysis on Self-Control in Intertemporal Choice and Mediterranean Dietary Pattern

**DOI:** 10.3389/fpubh.2019.00165

**Published:** 2019-06-26

**Authors:** Brian C. Howatt, María José Muñoz Torrecillas, Salvador Cruz Rambaud, Taiki Takahashi

**Affiliations:** ^1^Department of Psychological Sciences, Kansas State University, Manhattan, KS, United States; ^2^Department of Economics and Business, Universidad de Almería, Almería, Spain; ^3^Department of Behavioral Science, Center for Experimental Research in Social Sciences, Hokkaido University, Hokkaido, Japan

**Keywords:** Mediterranean diet, intertemporal choice, time discounting, impulsivity, self-control

## Abstract

This paper completes Muñoz Torrecillas et al. (1) results and conclusions investigating the relationship between adherence to healthy dietary habits, specifically the Mediterranean Diet (hereinafter, MD), and impulsivity in intertemporal choices. Impulsivity can be defined as the strong preference for small immediate payoffs over larger delayed payoffs, and in the original study this behavior was captured by the parameter *k* (discount rate of the hyperbolic discount function), calculated using an automated scoring mechanism. Adherence to MD was measured by the KIDMED index and then grouped into three levels: high, medium, and low. While the authors observed that individuals in the high adherence group had the shallowest discounting and individuals in the low adherence group had the steepest discounting, the data were not statistically analyzed in depth. Therefore, the purpose of the present paper is to propose a preliminary quantitative model for this relationship and evaluate its significance. Tests revealed a significant interaction between adherence to MD and magnitude of delayed rewards when predicting discount rates. Specifically, the degree to which impulsivity decreases as adherence to MD increases is strongly influenced by delayed rewards of smaller magnitude. These findings are consistent with the authors' claims that healthy dietary habits may be closely linked with greater self-control when payoffs are small, and thus warrant further examination. The results do not indicate causality though, so future studies could also investigate the directions of this relationship as a means of developing behavioral interventions.

## Introduction

Dietary habits are an integral part of our everyday life, and the quality of one's diet can significantly impact their health. For example, adhering to the Mediterranean Diet (MD), which is richly balanced in fruits, vegetables, and fish, has been associated with greater longevity and a decreased risk of metabolic syndrome, cardiovascular and/or cancer mortality and neurodegenerative diseases (2–4). However, global consumption patterns have increasingly shifted toward cheaper, heavily-processed foods, and beverages that are low in crucial nutrients but high in sugar and fat, raising alarms in public health communities around the world (5, 6). Although the influence of diet on health has been and continues to be extensively covered, what is less known is how these habits might affect everyday behavior.

Recently, research has linked clinical eating disorders (e.g., obesity and bulimia) to reduced self-control, suggesting that a degraded quality of diet (and subsequently, life) is related to overly impulsive behavior (7–10). If an individual is highly impatient, they may prefer more immediately gratifying, but unhealthy foods over taking the time to prepare, and benefit from, healthier meals. Moreover, they may eschew eating or retaining their intake in order to achieve a particular body image at the expense of long-term health.

The inverse may also hold true, though. Steele et al. (11) demonstrated that rats fed diets high in fat and sugar made substantially more impulsive choices than a separate cohort of rats fed a standard chow diet. Interestingly, Steele et al. (11) then had the rats with the high-fat and high-sugar diets switch back to a standard chow diet, and observed a significant decrease in their impulsive choices. These results indicate that diet can directly influence behavior, so this relationship may exhibit highly cyclic tendencies.

Therefore, a closer investigation into how an individual's dietary intake and economic decision-making influence each other is crucial to accomplishing the goals of public health policies. Muñoz Torrecillas et al. (1) set out to explore this relationship in people by testing whether greater adherence to MD was associated with lower impulsivity. And if such a meaningful connection was found, then subsequent research could examine this association within a causal scope, possibly leading to the development of interventions prescribed to promote improvements in one's diet and self-control.

**Figure 1:**
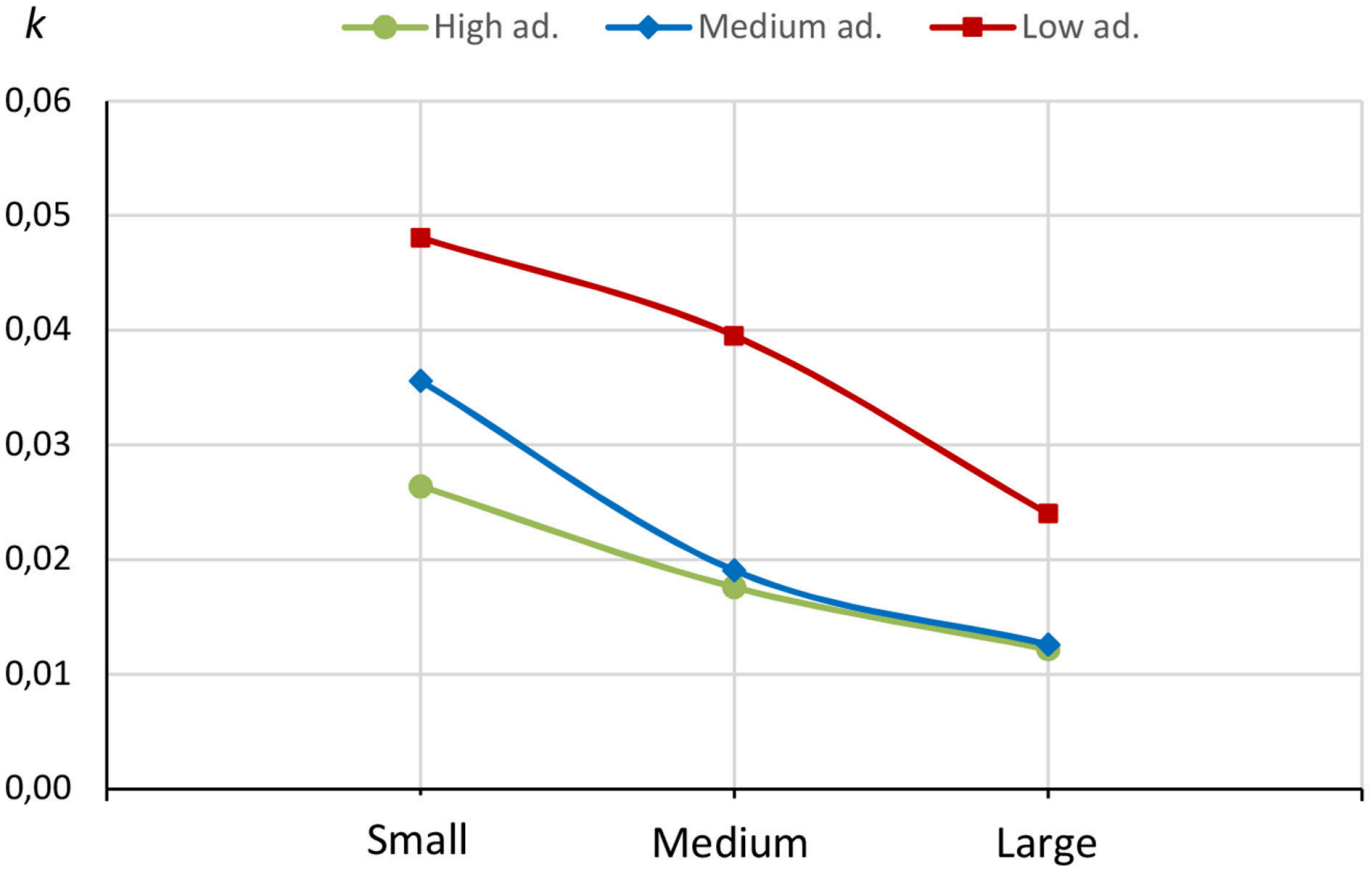
Raw *k*-values for small, medium, and large delayed rewards, by the three diet adherence groups. Original graph from Muñoz Torrecillas et al. (1).

Muñoz Torrecillas et al. (1) observed that participants in the highest diet adherence group exhibited the lowest impulsivity, and participants in the lowest diet adherence group displayed the greatest impulsivity (see Figure 1; larger *k*-values represent greater impulsivity), but these differences were not statistically analyzed. Therefore, the purpose of the present paper is to quantitatively model the relationship between dietary adherence and impulsivity, and put forth preliminary evidence regarding its nature.

## Materials and Methods

The original experiment used two different questionnaires to obtain information regarding participants' dietary adherence patterns and impulsive inclinations. First, the KIDMED test (12) provides an index of adherence to the Mediterranean Diet, and second, Kirby et al.'s (13) intertemporal choice questionnaire for inferring discount rates. Demographic information was also collected but not included in the current analysis.

### KIDMED Index and Intertemporal Choice Questionnaire

The KIDMED test devised by Serra-Majem et al. (12) provides a degree of adherence to MD by requesting answers in the affirmative or negative to 16 questions regarding food habits [see (1), Supplementary Material]. Answers congruent with MD principles are assigned a value of +1, and those incongruent, a value of −1; these values are then summed, with higher total scores implying greater adherence to MD. For this particular sample, a range from −1 to 12 was observed. Typically, these scores are then used to classify participants according to distinct adherence groups with pre-established thresholds: low, medium, or high (1, 12). However, creating arbitrary splits in scale data can lead to a loss of information and statistical power (14, 15), so in this analysis KIDMED scores were treated as a continuous measure.

Impulsivity was measured by means of the *k*-parameter (discount rate) in the hyperbolic discount function (16):

$$\begin{array}{l} \text{SIR}=\frac{\text{LDR}}{1+kd},\;k>0, \end{array}$$

where SIR is the smaller immediate reward, LDR is the larger delayed reward, and *d* is the delay until the receipt of LDR. The larger *k* is, the more heavily participants devalue future rewards and, thus, the more impulsive they are implied to be.

Kirby et al.'s (13) 27-item monetary choice questionnaire [see (1), Supplementary Material] was used to obtain participants' preferences between a series of SIRs and LDRs at various delays. Additionally, there were three levels of LDR size: small (from $25 to $35), medium (from $50 to $60), and large (from $75 to $85), each consisting of nine choice responses. Muñoz Torrecillas et al. (1) then calculated participants' *k*-values for each of the three magnitude groups, as well as an overall estimate, from this response data using an automated scoring mechanism developed by Kaplan et al. (17, 18). Lastly, because raw *k*-values tend to be highly skewed, the natural log transformation of these estimates were used in the analysis.

### Sample

The original sample consisted of 207 students at the Business School of the University of Almería (Spain), who voluntarily participated in answering the questionnaires. Almería is a Mediterranean province in the Southeast of Spain, a country that traditionally has followed a Mediterranean diet.

Eleven questionnaires were dropped due to incomplete surveys and inconsistent responding for a total of 196 participants. For the present analysis, one additional participant was dropped due to inconsistent responding (see Appendix A), so the final sample was 195. Regarding the composition of our sample, 55% of the participants were men and 45% women, and the mean age was 22 years.

### Procedure

Students were informed, before answering the questionnaires, that these will be voluntary and anonymous. After collecting the data, the KIDMED scores and the discount rates (*k*-values) were calculated for each individual, as explained in Muñoz Torrecillas (1).

To test the hypothesis that adherence to MD and rates of discount are related, a multilevel linear regression was run in JMP Pro V.13 statistical software with *log k-values* regressed on mean-centered *Adherence* scores, *LDR Magnitude*, and their interaction. *Subjects* was included as a random effect to allow the model intercept to vary across participants.

The primary advantage of using a multilevel model in this analysis is that it can correctly treat LDR magnitude as repeated-measures type data, while standard linear regression cannot (19). Another benefit is that the individual differences in *k*-values across the magnitude groups can be modeled in relation to the rest of the sample. This reduces the bias typically observed when averaging across groups, and also helps to diminish the impact of high leverage data by shrinking it closer to toward the mean.

## Results

Results yielded a significant main effect of *Magnitude, F*_(2, 386)_ = 141.11, *p* < 0.001, and a significant interaction effect of *Adherence* and *Magnitude, F*_(2, 386)_ = 3.54, *p* = 0.03. However, there was no significant main effect of *Adherence, F*_(1, 193)_ = 2.45, *p* = 0.12. These results suggest that the relationship between MD adherence and impulsivity is differentially influenced by the magnitude of the delayed reward. When delayed rewards are relatively small, participants exhibited a greater level of impulsivity. Conversely, when delayed rewards are relatively large, participants exerted greater self-control and were much more willing to wait for them. Table 1 presents the unstandardized main effect slope estimates and 95% confidence intervals.

**Table 1:** Main effect parameter estimates of dietary adherence and LDR magnitude predicting log *k*-values.

	**B**	**95% *CI***	***t***	***p***
Intercept	−4.58	[−4.75, −4.41]	−52.68	<0.001
MD adherence	−0.05	[−0.13, 0.02]	−1.56	0.12
Small LDR	0.56	[0.49, 0.64]	14.21	<0.001
Medium LDR	0.02	[−0.05, 0.10]	0.66	0.51
Large LDR	−0.59	[−0.67, −0.51]	−14.87	<0.001

*Adherence was centered by subtracting 6.25. LDR Magnitude was effect coded as [+1 = Small, 0 = Medium, −1 = Large]*.

**Figure 2:**
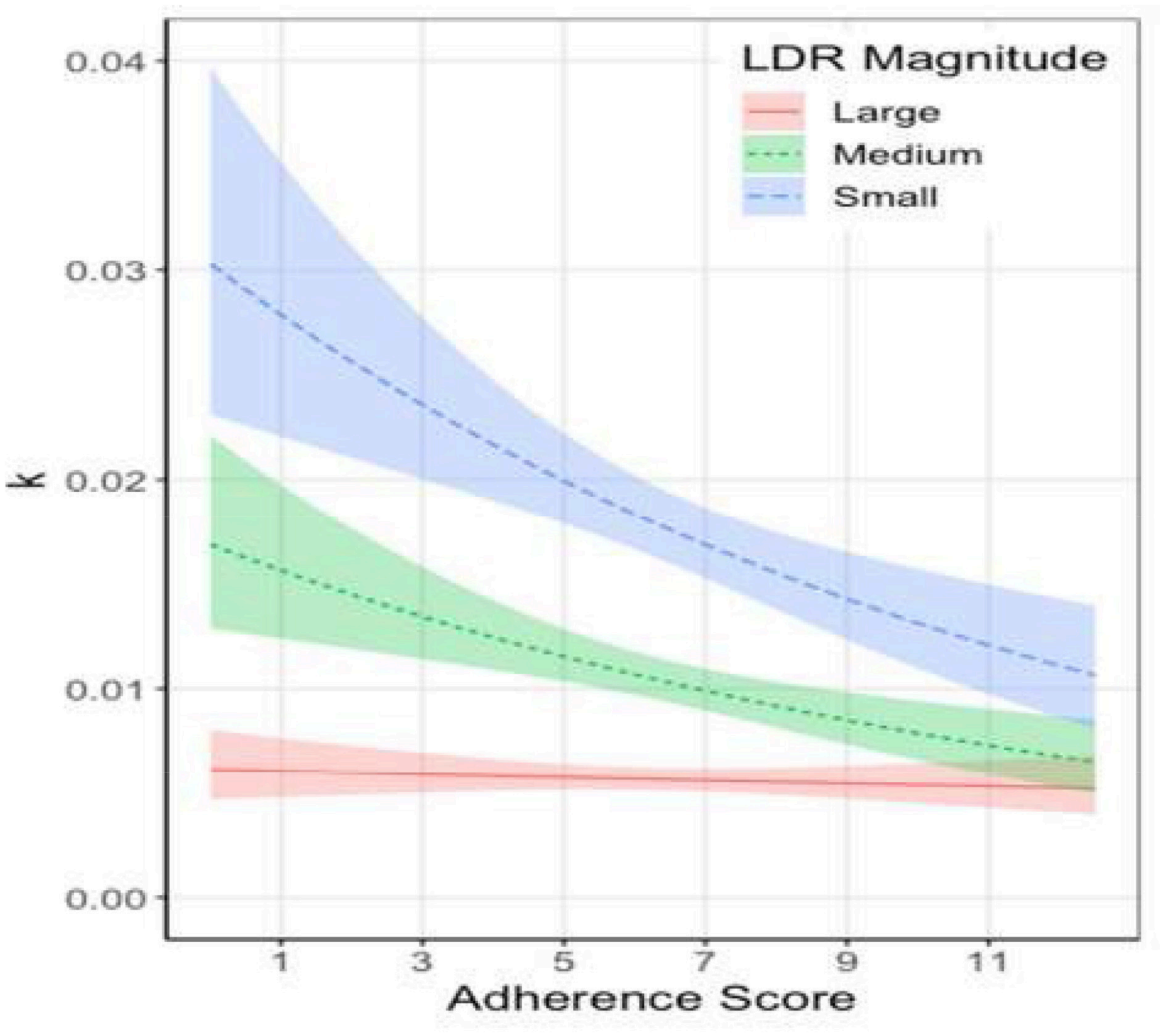
Model predicted raw *k*-values of each LDR magnitude by Adherence to MD scores. Shaded areas represent 95% confidence intervals.

Interaction tests using JMP Pro's custom test feature revealed that the slope of the small LDR was significantly less than zero, the slope of the medium LDR was marginally less than zero, and the slope of the large LDR was not significantly different from zero. These results suggest that the rate of impulsivity significantly decreases for relatively small rewards as MD adherence increases. Furthermore, the rate of impulsivity slightly decreases for relatively medium-sized delayed rewards as MD adherence increases. However, the rate of impulsivity stayed fairly constant for relatively large delayed rewards as MD adherence increased. Table 2 provides the unstandardized slope estimates of the interactions and 95% confidence intervals. Figure 2 shows the model predictions back-transformed into the original scale.

**Table 2:** Parameter estimates of the interaction effects predicting log *k*-values.

	**B**	**95% *CI***	***t***	***p***
Small LDR·adherence	−0.08	[−0.16, −0.01]	−2.06	0.04
Medium LDR·adherence	−0.07	[−0.15, 0.01]	−1.88	0.06
Large LDR·adherence	−0.01	[−0.09, 0.07]	−0.32	0.75

## Discussion

The results of the statistical analysis provide evidence for an inverse relationship between an individual's adherence to the Mediterranean Diet (MD) and impulsivity when delayed rewards are of smaller magnitude. Although a decline in impulsivity for delayed rewards of larger magnitude was not observed with greater MD adherence, participants did on average exhibit significantly greater self-control for these payoffs. These findings are consistent with (1) original claims, as well as other established research demonstrating smaller rewards are discounted more steeply (20–22) and unhealthy diets are related to greater impulsivity (9, 10). Therefore, dietary adherence and discount rates could potentially be used in conjunction with one another to determine at-risk populations in which to target health policies.

It is interesting to note that (23) also reported that discount rates for smaller sized rewards was the most sensitive to the medical treatment and abstinence in alcoholic patients over 2 months. This indicates that magnitude effect exists not only in discount rates *per se*, but also in the sensitivity to the manipulation of food and drug intake or the strength of relationship between impulsivity and (un)healthy habits.

One limitation of this analysis is that the KIDMED index has multiple dependencies between questions. For example, Question 1 asks whether the participant consumes a piece of fruit or fruit juice every day, and Question 2 asks whether the participant has a second piece of fruit every day. If a participant does not consume one serving of fruit every day, then they cannot consume additional servings. Thus, the answer to Question 2 is highly dependent on the answer to the first question. This violates the “independence of observations” assumption of linear regression and can bias the model fit (24). Using another scale (or modifying the KIDMED test) with questions that are completely independent from each other may provide a more informative index of adherence to MD and bolster its predictive qualities.

A second limitation is that while the Kirby et al. (13) questionnaire (and others similar to it) is popular in behavioral experiments due to its convenience, such highly stylized designs often struggle with ecological validity. Operant tasks analogous to non-human animal procedures, in which subjects experience and learn from the delay and payoffs in real time, may provide greater utility for evaluating impulsive behavior in humans (25–28). And previous studies have reported substantial improvements in discounting tendencies when using naturalistic approaches to capture this behavior (29–33). An added benefit of these types of naturalistic methods is that time and diet based interventions, among others, can be developed around them to promote self-control through desensitizing subjects to delays (34) or engaging in healthier consumption habits (11).

Muñoz Torrecillas et al. (1) set out to build an empirical foundation regarding the association between dietary habits and intertemporal choices, a relationship that has important implications for both individual behavior and global policy-making. The results of this present analysis support their hypothesis that adherence to a healthy diet and greater self-control are connected. Therefore, future research could continue to expand on this work by analyzing and quantifying if people controlling their life style and dietary habits may also be controlling impulsivity, and studying the efficacy of interventions to enhance the quality of life in both clinical and non-clinical populations.

## Data Availability

Publicly available datasets were analyzed in this study. This data can be found here: a “https://www.ncbi.nlm.nih.gov/pmc/articles/PMC6013565/.”

## Ethics Statement

Ethical review and approval was not required for this study in accordance with the national and institutional requirements. The University of Almería approved the collection of data among students who voluntarily agreed to answer the anonymous questionnaire.

## Author Contributions

MM, SC, and TT contributed conception and design of the study and collected the data and organized the database. BH performed the statistical analysis and wrote the first draft of the manuscript. MM and SC funding acquisition. All authors contributed to manuscript revision, read, and approved the submitted version.

### Conflict of Interest Statement

The authors declare that the research was conducted in the absence of any commercial or financial relationships that could be construed as a potential conflict of interest.
