# Supplementary material for: A New Analysis on Self-Control in Intertemporal Choice and Mediterranean Dietary Pattern
Source: Front Public Health. 2019 Jun 26;7:165. doi: 10.3389/fpubh.2019.00165 (PMC6611428; doi:10.3389/fpubh.2019.00165)
Supplement: Supplementary file 1 [file Data_Sheet_1.docx]

**Appendix A**

Kaplan et al’s (17) automated scoring mechanism provides a consistency index that calculates “the degree to which the respondents’ selections are consistent with response patterns preceding, as well as following, the switch(es) from SIR choices to LDR choices” ((17), p. 298,). Higher consistency thus implies that if a participant prefers the SIR to the LDR at a particular *k*-value, they will also prefer the SIR for the remaining items with equal or greater *k*-values. This is a theoretically sensible exclusion criterion, as participants should exhibit relatively stable sensitivities to the rate of discount.

However, this measure of consistency is not applied to preferences across magnitudes. If a participant prefers the LDR to the SIR at a particular *k*-value and delay, then, theoretically, they should also prefer the LDR at the same *k* and delay for items when the LDR is greater. Subject 29 did not demonstrate this magnitude consistency. Subject 29 only preferred the LDR to the SIR once throughout the entire 27-item survey, when the *k* was at its largest value and the LDR was classified as ‘small’ magnitude. They did not prefer the LDR at the exact same *k*-value and delay when the LDR was classified as either ‘medium’ or ‘large’ magnitude. This would be analogous to preferring $20 to $10 in 7 days, but preferring $40 to $80 in 7 days, and implies that large rewards are discounted more steeply than small rewards. This is not theoretically consistent, and for that reason Subject 29’s data was excluded from the final analysis.
